# Cuprizone demyelination induces a unique inflammatory response in the subventricular zone

**DOI:** 10.1186/s12974-016-0651-2

**Published:** 2016-08-22

**Authors:** James M. Hillis, Julie Davies, Mayara Vieira Mundim, Osama Al-Dalahmah, Francis G. Szele

**Affiliations:** 1 Department of Physiology, Anatomy and Genetics, University of Oxford, South Parks Road, Oxford, OX1 3QX UK; 2 Department of Biochemistry, Universidade Federal de São Paulo, São Paulo, 04039-032 Brazil

**Keywords:** Subventricular zone, Multiple sclerosis, Inflammation, Galectin-3, Corpus callosum, Demyelination

## Abstract

**Background:**

Cuprizone leads to demyelination of the corpus callosum (CC) and activates progenitor cells in the adjacent subventricular zone (SVZ), a stem cell niche which contributes to remyelination. The healthy SVZ contains semi-activated microglia and constitutively expresses the pro-inflammatory molecule galectin-3 (Gal-3) suggesting the niche uniquely regulates inflammation.

**Methods:**

We studied the inflammatory response to cuprizone in the SVZ and CC in Gal-3 knockout mice using immunohistochemistry and with the in vitro neurosphere assay.

**Results:**

Cuprizone caused loss of myelin basic protein (MBP) immunofluorescence in the CC suggesting demyelination. Cuprizone increased the density of CD45+/Iba1+ microglial cells and also increased Gal-3 expression in the CC. Surprisingly, the number of Gal-3+ and CD45+ cells decreased in the SVZ after cuprizone, suggesting inflammation was selectively reduced therein. Inflammation can regulate SVZ proliferation and indeed the number of phosphohistone H3+ (PHi3+) cells decreased in the SVZ but increased in the CC in both genotypes after cuprizone treatment. BrdU+ SVZ cell numbers also decreased in the SVZ after cuprizone, and this effect was significantly greater at 3 weeks in *Gal-3*^*−/−*^ mice compared to WT, suggesting Gal-3 normally limits SVZ cell emigration following cuprizone treatment.

**Conclusions:**

This study reveals a uniquely regulated inflammatory response in the SVZ and shows that Gal-3 participates in remyelination in the cuprizone model. This contrasts with more severe models of demyelination which induce SVZ inflammation and suggests the extent of demyelination affects the SVZ neurogenic response.

**Electronic supplementary material:**

The online version of this article (doi:10.1186/s12974-016-0651-2) contains supplementary material, which is available to authorized users.

## Background

Multiple sclerosis (MS) is a debilitating neurological condition resulting from immune-mediated demyelination [1]. Various etiologies increase the risk of developing MS, including genetic and environmental origins, but the exact causes are unclear [2]. Several different murine models exist that partially recapitulate different aspects of MS and demyelination [3]. Cuprizone is a copper-chelator that impacts cell metabolism and leads to demyelination, and if continued, eventual oligodendrocytic and neuronal death. Cuprizone is administered via food for a defined time period before returning the mice to standard chow to allow remyelination to occur. While the cuprizone model maintains an intact blood-brain barrier with no T cell infiltration, it extensively activates microglia and macrophages [4–6]. The cuprizone model is increasingly used because of its reversibility and reproducibility, reviewed in [7, 8].

Cuprizone treatment leads to demyelination in the corpus callosum (CC). The CC in turn forms the roof of the subventricular zone (SVZ), a neural stem cell niche that generates neurons and glia throughout life [9]. The cuprizone model therefore enables investigation into the SVZ response to demyelination and remyelination in the CC. A recent fate-mapping study demonstrated that SVZ cells contributed to remyelination in the CC after cuprizone-induced demyelination [10]. These cells produced thicker myelin than oligodendrocytes derived from local oligodendrocyte progenitor cells (OPC) [10]. These findings are consistent with previous research showing SVZ cells can differentiate into oligodendrocytes constitutively as well as after demyelination [11–13] and indicate SVZ cells migrate to the demyelinated CC in MS [13]. OPCs may have broader reparative effects throughout the CNS than SVZ cells, but SVZ-derived progenitors contribute to repair around the lateral ventricles. This is clinically relevant since it has been known for close to a century that MS lesions frequently emanate from the ventricular surface [14].

The SVZ thus provides a conveniently positioned nest of stem cells that could benefit MS. Interestingly, data from the past several years suggest inflammation is uniquely regulated in the SVZ, often exhibiting features dramatically different to the CC or other nearby regions. We found that even in the absence of injury, CD45 levels and microglial proliferation were significantly higher in the SVZ than in adjacent regions [15]. We recently showed the SVZ expresses higher levels of chemokines than the nearby cerebral cortex [16]. These chemokines rose markedly in the SVZ after Theiler’s murine encephalomyelitis virus (TMEV) injections which induce demyelination. However loss of Gal-3 blocked these increases, suggesting a pivotal role in the inflammatory response in the TMEV model of demyelination [16]. Perhaps because of these differences, the SVZ can exhibit inflammation in response to brain insults unlike adjacent regions. Traumatic brain injury induced massive inflammation in the CC, but all manifestations of inflammation remained stable in the SVZ [15]. In contrast, TMEV specifically targeted the SVZ and led to greater inflammation there compared to surrounding regions [16, 17]. We had reported in a preliminary study that microglial density was maintained in the SVZ while rising in the CC after cuprizone [17]. We therefore asked if inflammation was differentially regulated in the SVZ compared to the CC after cuprizone treatment. Initial studies suggested that inflammation can be detrimental to neurogenesis and brain repair [18, 19]. Further studies have shown however that microglia can enhance neurogenesis in vitro and in vivo [20, 21]. Thus, it is important to determine context-dependent inflammation in the SVZ and how this affects the reparative potential of this largest pool of endogenous brain stem cells.

Another clue that inflammation is uniquely regulated in the SVZ is that the pro-inflammatory protein galectin-3 (Gal-3) is homeostatically expressed in the SVZ and RMS although elsewhere in the central nervous system it is only detectable after injury [22]. Gal-3 has been implicated in MS; it is expressed at high levels in active lesions around the lateral ventricles, and it modulates animal models of MS [16, 23]. Gal-3 was initially termed Mac-2 because it was identified in activated macrophages [24]. However, in the SVZ, it is expressed by astrocytes and ependymal cells and serves to maintain speed and rostral directionality of SVZ neuroblast migration, thereby positively contributing to olfactory bulb neurogenesis rates [22]. Gal-3 expression increases leukocyte infiltration into the SVZ in the EAE and TMEV demyelination models, leading to a worse phenotype [16, 25]. Gal-3 has also been reported to increase oligodendrocyte differentiation in vitro and enhance myelination in vivo [26] and loss of Gal-3 inhibited oligodendrocyte maturation and microglial activation after cuprizone [27]. Here, we hypothesized that cuprizone-induced demyelination would cause SVZ inflammation and that Gal-3 would regulate SVZ progenitor after cuprizone treatment.

## Methods

### Animals

129Sv wild-type mice (*Gal-3*^*+/+*^) were obtained through the University of Oxford Biomedical Services Specific Pathogens Free Breeding Unit. Gal-3 knockout mice on a 129Sv background (*Gal-3*^*−/−*^) were obtained from Françoise Poirier’s laboratory (Institut Jacques Monod, Paris; Colnot et al., 1998). *Gal-3*^*+/+*^ and *Gal-3*^*−/−*^ mice were bred to produce heterozygote mice (*Gal-3*^*+/−*^), which were subsequently mated with one another to produce *Gal-3*^*+/+*^ and *Gal-3*^*−/−*^ littermate controls. For experiments using adult animals, these animals were used. For experiments using postnatal animals, the littermate controls were bred with other mice of the same genotype to produce entire litters of *Gal-3*^*+/+*^ or *Gal-3*^*−/−*^ pups. Animals were maintained in individually ventilated cages on 12-h light/dark cycles with free access to food and water. Procedures were performed with University of Oxford Research Ethics Committee approval in accordance with the Animals (Scientific Procedures) Act of 1986 (UK). All efforts were made to minimize animal suffering and distress.

### Bromodeoxyuridine injections

To create label retaining SVZ cells, bromodeoxyuridine (BrdU) was administered via intraperitoneal (IP) injection at 50 mg/kg. It was given in six 12-hourly doses during the first 3 days of cuprizone administration.

### Cuprizone administration

Cuprizone was administered at 0.2 % to 8-week-old male and female mice ad libitum in chow (International Product Supplies). Treated mice received cuprizone chow for 3 or 6 weeks to cause demyelination, or 6 weeks followed by 10 days of control chow to induce remyelination. Control mice received the same chow without cuprizone. The 6-week cuprizone period was chosen as oligodendrocytes decrease greatly by that time, while the speed of oligodendrogenesis should have peaked after ~10 days of remyelination [28].

### Fluorescent immunohistochemistry

Brains for immunohistochemistry were removed following IP pentobarbitone overdose and transcardiac perfusion with cold normal saline and 4 % paraformaldehyde (PFA). They were postfixed in 4 % PFA overnight and then cryoprotected in 30 % sucrose in 0.1 M phosphate buffer. They were frozen in dry ice, stored at −80 °C and then sectioned into 30-μm coronal sections using a freezing microtome. Fluorescent immunohistochemistry was performed as described previously [29]. Primary antibodies are outlined in Table 1. BrdU antigen retrieval was achieved with 1 M HCl at 38.5 °C for 1 h.

**Table 1:** Antibodies used in study

Antigen	Dilution	Host	Manufacturer	Product #
BrdU	1:500	Sheep	Abcam	ab1893
CD45	1:500	Rat	Chemicon	CBL1326
Dcx	1:100	Goat	Santa Cruz	sc-8066
Gal-3	1:100	Rabbit	Santa Cruz	sc-20157
Gal-3	1:100	Rat	Santa Cruz	sc-23938
GFAP	1:400	Rat	Life Technologies	13-0300
Iba1	1:400	Goat	Abcam	Ab5076
Il1-β	1:100	Rabbit	AbD Serotec	AAM13G
Mash1	1:200	Mouse	BD Pharmingen	556604
MBP	1:100	Goat	Santa Cruz	sc-13914
Olig2	1:1000	Rabbit	Chemicon	AB9610
PHi3	1:400	Rabbit	Upstate	06-570

### Microscopy

Epifluorescence images were obtained using a Leica DMIRB microscope with a Hamamatsu C4742-95 digital camera or a Leica DMR microscope with a Leica DFC-500 digital camera. Images were acquired in Openlab software (Improvision) and processed in Volocity 4 software (PerkinElmer), or acquired in Leica Firecam 3.4.1 software. Confocal images were obtained on a Zeiss LSM 710 laser scanning confocal microscope using the Z-stack and tile functions as appropriate. They were analyzed in LSM Image Browser 4.2 (Zeiss). The majority of imaging, including all images for threshold analysis, used constant camera settings within a given experiment.

### Image analysis

All image acquisition, analysis and quantification, was performed blinded to the genotype and treatment, except for the initial acquisition of CC and cortical myelin basic protein (MBP) images. The sections analyzed were between Bregma 0.6 and 0.2 mm. The most caudal sections where just anterior to crossing of the anterior commissure. Live counting was used for BrdU+ and phosphohistone H3 (PHi3+) cells in the SVZ and CC, and Olig2+ cells in the SVZ. Sample images were acquired of other cell markers and then counted using ImageJ. Macros were developed that displayed only the DAPI channel, so that the appropriate region could be selected and cropped without the bias of seeing other channels. The macros then switched the view to other channels for cell counting. For MBP threshold analysis, regions were initially selected using only the DAPI channel. Histograms for fluorescence intensity levels between 0 and 255 were then obtained. Microsoft Excel was used to plot the intensity distribution and select an appropriate threshold above which MBP labeling was considered positive. The percentage of pixels with intensity greater than the threshold value was then calculated.

### Real-time polymerase chain reaction

SVZ tissue from individual 6-week *Gal-3*^*+/+*^ and *Gal-3*^*−/−*^ mice was microdissected in Hanks’ Buffered Saline Solution (HBSS). The sample was centrifuged to pellet tissue and aspirate excess HBSS. It was snap frozen in liquid nitrogen and stored at −80 °C. RNA extraction was performed using an RNeasy Mini Kit. Before extraction, individual samples were pooled to provide three groups of four animals for each genotype. All samples provided a 260/280 ratio between 2.08 and 2.10, which was considered to provide satisfactory RNA quality. Reverse transcription was performed using a High Capacity RNA to cDNA Kit (Applied Biosystems). Quantitative PCR (qPCR) used TaqMan Gene Expression Assays (Life Technologies) and TaqMan Gene Expression Master Mix (Life Technologies). It was performed on a 7900HT Fast Real-Time PCR System (Applied Biosystems) using technical triplicates and standard curves and following the TaqMan Gene Expression Master Mix Protocol for 384-well plates. Data was initially analyzed using SDS Software v2.4, and aberrant replicates were removed. EGFr and B2m had efficiency between 90 and 110 % and *R*^2^ ≥ 0.998 while CCL2 had *R*^2^ = 0.965, Ccr2 *R*^2^ = 0.948, and Mmp9 *R*^2^ = 0.987 (Egfr Mm00433023 m1; Ccl2 Mm00441242 m1; Ccr2 Mm00438270 m1; Mmp9 Mm00442991 m1; B2m Mm00437762 m1). Galectin-1 (Lgals1) Mm00839408 g1; Galectin-3 (Lgals3) Mm00802901 m1; Galectin-8 (Lgals8) Mm01332239 m1; Galectin-9 (Lgals9) Mm00495295 m1. Data were exported to Microsoft Excel, where individual gene quantities were normalized to B2m and divided by *Gal-3*^*+/+*^ quantities to create fold-change ratios. Statistics were performed on normalized values using a Student’s *t* test as described later.

### Neurosphere proliferation assay

Primary SVZ cultures were obtained from P4-5 *Gal-3*^*+/+*^ and *Gal-3*^*−/−*^ mice. Animals were sacrificed using anesthesia (hypothermia) followed by decapitation. Whole brains were dissected then cut into 500-μm coronal sections using a McIlwain tissue chopper. SVZ was microdissected in HBSS and stored on ice until dissection was complete. Cells were dissociated mechanically with fine iris scissors and enzymatically through Accutase incubation (Sigma, A6964) for 10 min at 37 °C. Cells were then washed three times in Neurobasal A+ (1×, B27 1×, glutamine 2 mM and penicillin/streptomycin 50k U/L) including centrifugation for 5 min at 1200 rpm for each wash, suspended in NB-A+ with growth factors (EGF and FGF-2, 20 ng/ml, R&D Systems), counted, and seeded in 96-well plates at 5 × 10^4^/mL of NB-GF. Cultures were incubated at 37 °C with 5 % CO_2_ for 7 days before whole wells were imaged using an AMF4300 EVOS® FL Imaging System (Life Technologies). Neurospheres were aspirated and then dissociated enzymatically, washed, suspended, counted, and seeded at 5 × 10^3^/mL of NB-GF. Cultures were then incubated for a further 7 days before imaging of secondary neurospheres. Images were analyzed in ImageJ using the Stack Sorter plugin to merge images (http://www.optinav.com/Stack-Sorter.htm).

### Statistics

Data from the appropriate quantification was collated into Microsoft Excel. It was then analyzed using scripts written for SPSS 21. For experiments comparing one factor between two groups, a Student’s *t* test was performed. For experiments comparing one factor between three or more groups, a one-way ANOVA was performed. For experiments comparing two factors, a two-way ANOVA was performed. For experiments comparing two factors for a variable quantified in multiple SVZ or CC anatomical regions, a repeated-measures ANOVA was performed. Bonferroni post hoc tests were used to determine significant pairwise comparisons in ANOVAs. Graphs were produced in Prism 5 (GraphPad) and show mean ± standard error of the mean (SEM).

## Results

**Fig. 1:**
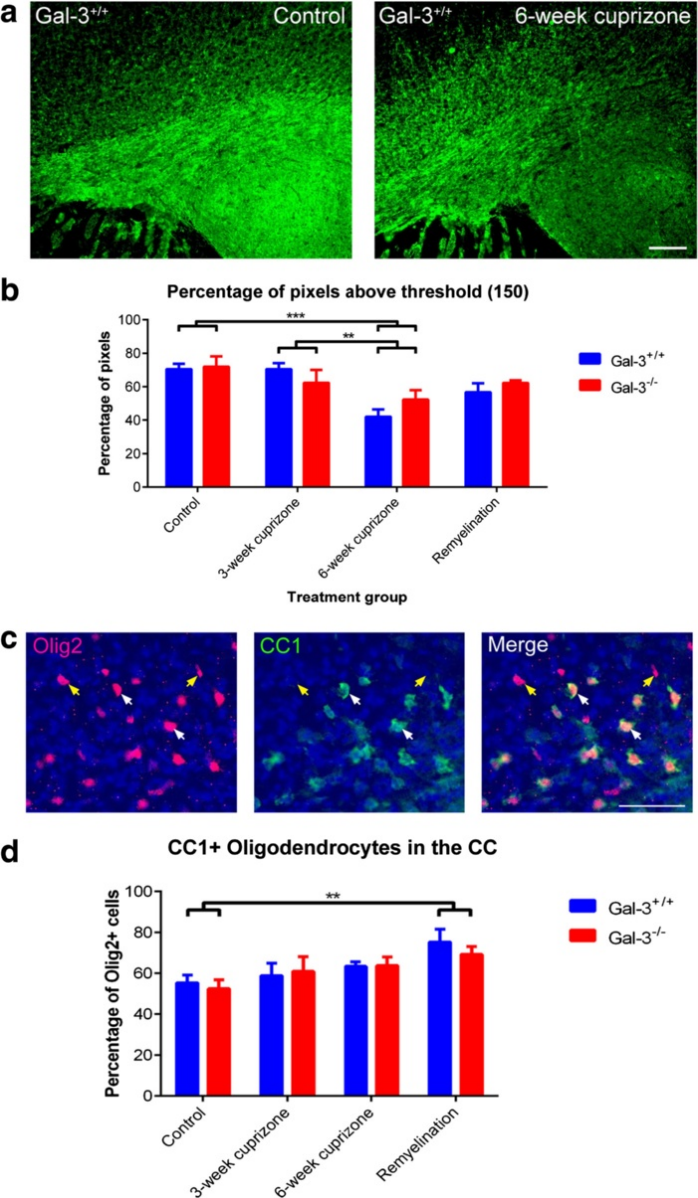
MBP levels decrease in the CC after cuprizone treatment and effects of Gal-3 on CC1+ oligodendrocytes. **a** Representative images of MBP immunohistochemistry in controls and after demyelination of *Gal-3* ^*+/+*^ mice. *Scale bar* 100 μm. **b** Graph showing percentage of pixels above the MBP threshold of 150. Statistics calculated as two-way ANOVA with *N* = 6. Graphs show mean ± SEM. **p < 0.01, ***p < 0.001. **c** Immunohistochemistry showing co-localization of CC1 and Olig2. *White arrows*: CC1+/Olig2+. *Yellow arrows*: CC1-/Olig2+. *Scale bar* 50 μm. **d** Percent of Olig2+ cells that are CC1+. Statistics were calculated as two-way ANOVA with *N* ≥ 6. Graphs show mean ± SEM. **p ≤ 0.01

We began by confirming demyelination in the CC, using MBP immunohistochemistry to assess myelin levels. As previously described, MBP is one of the key proteins associated with the myelin sheath [30, 31] and can be used to assess demyelination and remyelination [27]. Gal-3 can affect the inflammatory response in multiple models of disease and injury [27, 32]. We did not detect differences in MBP immunofluorescence between *Gal-3*^*+/+*^ and *Gal-3*^*−/−*^ mice. The CC, however, showed decreased MBP immunofluorescence in both genotypes after cuprizone (Fig. 1a, b), confirming CC demyelination as has been reported [33, 34].

While the number or density of Olig2+ cells provides information on the number of oligodendrocyte-lineage cells, it does not indicate how mature these cells are. We therefore performed co-immunohistochemistry with the mature oligodendrocyte marker CC1 [35] (Fig. 1c). The labeling demonstrated a good overlap between CC1 and Olig2 immunofluorescence (Fig. 1c). The CC1+ cells were interpreted as mature, myelinating oligodendrocytes. The proportion of CC1+ oligodendrocytes increased with cuprizone treatment and then remyelination but was not affected by Gal-3 loss (Fig. 1d). The effect between treatment groups or interaction with genotype was not significant.

### The number of Gal-3+ cells in the SVZ decreased after cuprizone treatment

**Fig. 2:**
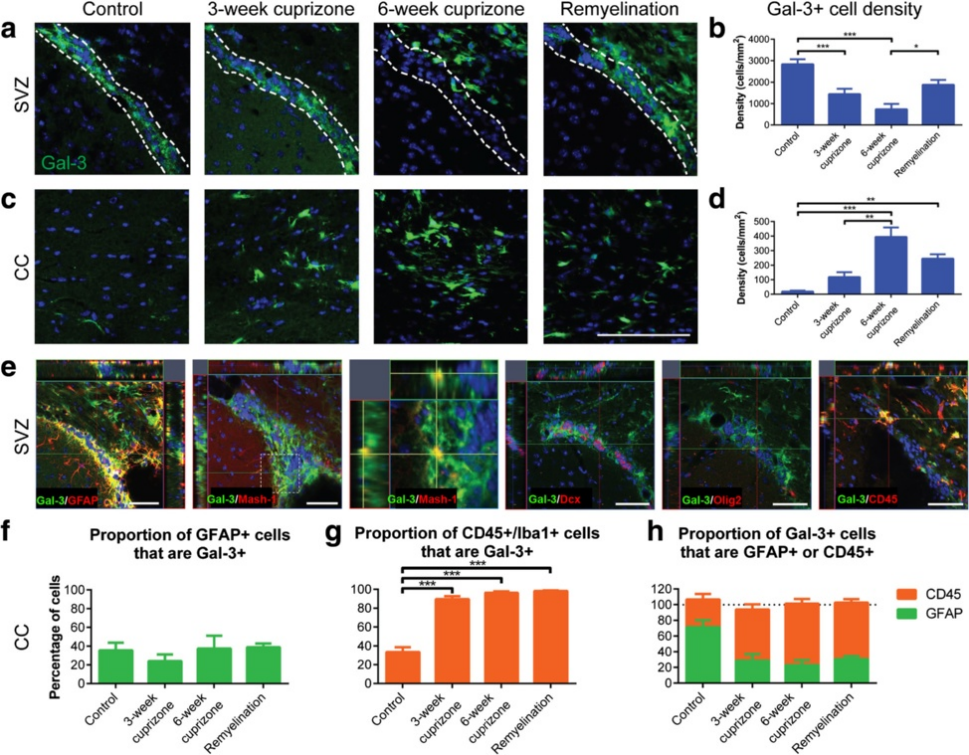
The number of Gal-3+ cells in the SVZ decreases after cuprizone treatment. **a**, **c** Representative images of Gal-3+ cells in the SVZ and CC after cuprizone treatment. *Scale bar* 100 μm. *White dotted lines* define SVZ boundaries. **b**, **d** Graphs demonstrating the density of Gal-3+ cells in the SVZ (**b**) and CC (**d**). Statistics calculated as one-way ANOVA with *N* = 4. Graphs show mean ± SEM. *p < 0.05, **p < 0.01, ***p < 0.001. **e** Representative images of the SVZ from 6-week cuprizone or remyelination brains showing Gal-3 (*green*) colocalization with GFAP+ astrocytes/neural stem cells, Mash-1+ TAPs and CD45+ hematopoietic cells (which includes CD45+/Iba1+ microglia), and lack of Gal-3 colocalization with Dcx+ neuroblasts and Olig2+ oligodendrocytes. We used *N* = 4 for all groups. *Dotted box* shows magnified region. *Scale bars* 50 μm. **f**–**h** Graphs showing the quantification of colocalization in the CC. Statistics calculated as one-way ANOVA with *N* = 4 for **f** and **g**. No statistics performed for **h**. Graphs show mean ± SEM. ***p < 0.001. Note that GFAP+ and CD45+ cells were quantified in different experiments, so aggregates in **h** will not necessarily equal 100 %

We previously showed that Gal-3 is selectively expressed in the SVZ and regulates SVZ inflammation after injections of the demyelinating virus TMEV [16, 22]. We therefore hypothesized that Gal-3 would have a role in the SVZ response to cuprizone-induced demyelination. Interestingly, the density of Gal-3+ cells decreased in the SVZ following cuprizone treatment (*N* = 4 per group; Fig. 2a, b). We included both dorsolateral SVZ astrocytes and ependymal cells in our quantification and noted that Gal-3 was lost from both cell types. The number of Gal-3+ cells/mm^2^ in control mice decreased significantly after 3-week cuprizone (*p* = 0.009 compared to control) and after 6-week cuprizone (*p* < 0.0005 compared to control). The density then increased in the remyelination treatment group (*p* = 0.034 compared to 6-week cuprizone). In contrast to the SVZ, Gal-3+ cell density increased in the CC of control mice significantly in the 6-week cuprizone group (*p* < 0.0005 compared to control and *p* = 0.002 compared to 3-week cuprizone) (Fig. 2c, d). The density then decreased in the remyelination group (*p* = 0.010 compared to control and *p* = 0.122 compared to 6-week cuprizone). These unexpected results showed that the regulation of Gal-3 expression after cuprizone was opposite between the SVZ and the CC, even though they are immediately adjacent.

We subsequently performed co-immunohistochemistry to determine the identity of Gal-3+ cells (Fig. 2e–h). They were mostly GFAP+ astrocytes or CD45+/Iba1+ microglia in both the SVZ and CC (*N* ≥ 4 for each group; Fig. 2e). Mash1+ cells correspond to neural lineage transit amplifying progenitor (TAP) cells in the SVZ [36]. Similar to what we had shown before [22], the occasional Mash-1+ TAP was also Gal-3+. There were very few Gal-3+/Dcx + neuroblasts (1 Gal-3+/Dcx + cell out of 337 Gal-3+ and 42 Dcx + cells counted in the CC) or Olig2+ oligodendrocytes (2 Gal-3+/Olig2+ cells out of 337 Gal-3+ and 436 Olig2+ cells counted in the CC). Interestingly, we found that the percentage of CD45+/Iba1+ microglia in the CC expressing Gal-3 increased significantly following cuprizone treatment (*p* ≤ 0.001 for control compared to each other group) (Fig. 2g). The percentage of astrocytes expressing Gal-3 remained stable (Fig. 2f). Overall, these data suggested that Gal-3 expression increased mostly on microglia in the CC after cuprizone.

### The subventricular zone contained fewer hematopoietic cells after cuprizone treatment

**Fig. 3:**
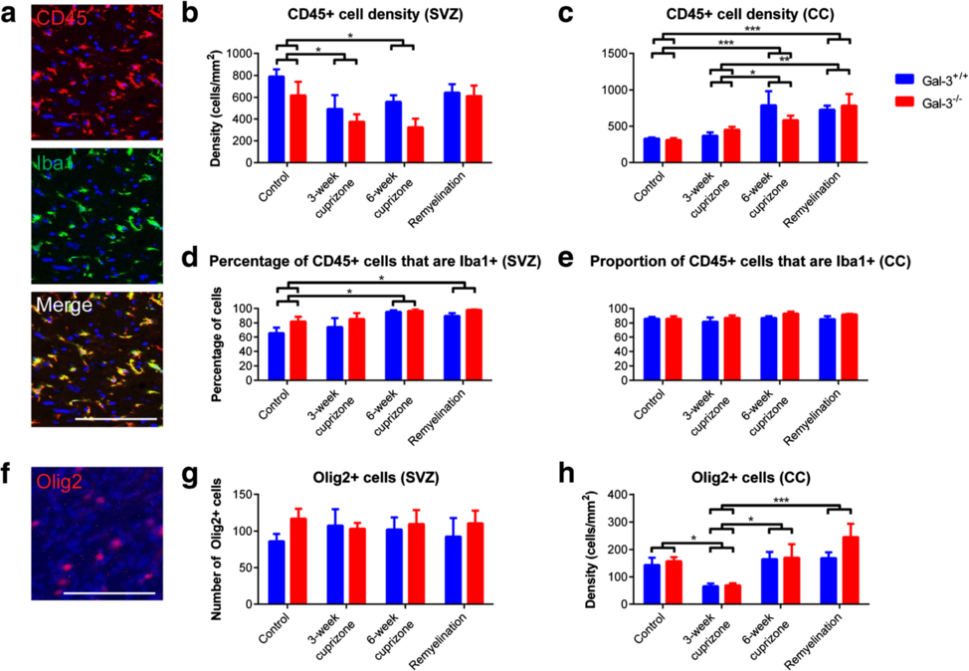
The number of CD45+ cells decreases in the SVZ after cuprizone treatment. **a** Representative images of CD45 and Iba1 immunohistochemistry in the CC. *Scale bar* 100 μm. **b**, **c** Graphs demonstrating the density of CD45+ cells in the SVZ and CC. **d**, **e** Graphs demonstrating the proportion of CD45+ cells that are Iba1+ in the SVZ and CC. Statistics calculated as two-way ANOVA with *N* < 5. Graphs show mean ± SEM. *p < 0.05, **p < 0.01, ***p < 0.001. **f** Representative image of Olig2+ immunohistochemistry from the CC of a WT remyelination mouse. **g**, **h** Graphs showing number of Olig2+ cells in the SVZ (**g**) and density in the CC (**h**). Statistics calculated using repeated-measures ANOVA for SVZ and two-way ANOVA for CC with *N* = 6 for each group. Graphs show mean ± SEM. *p < 0.05, ***p < 0.001

Previous work from our group showed that TMEV-induced demyelination targets inflammation to the SVZ [16, 17]. Here, we further investigated the density of CD45+ hematopoietic cells and CD45+/Iba1+ microglia in the SVZ and CC (Fig. 3a). The density of CD45+ hematopoietic cells decreased significantly in the SVZ yet increased in the CC during cuprizone treatment (*N* ≥ 6 per group; Fig. 3b, c). Although the density of CD45+ cells decreased in the SVZ after cuprizone, the proportion that were Iba1+ microglia was increased (Fig. 3d). This proportion, however, was similar across all conditions in the CC (Fig. 3e). There was no difference between Gal-3^+/+^ and Gal-3^−/−^ mice in the density of CD45+ cells nor the percentage of CD45+ cells that were Iba1+ in the SVZ and in the CC (Fig. 3b–e). Once again, the juxtaposition of CD45+ density between the SVZ and CC was noteworthy, as it suggested different inflammatory processes occurring in the two neighboring regions.

We examined potential genes that might have compensated for Gal-3 loss in the knockout mice and created a shortlist based on genes that differed between *Gal-3*^*+/+*^ and *Gal-3*^*−/−*^ mice in previous qPCR and proteomic arrays and genes thought to be regulated by Gal-3 which are relevant for SVZ biology [16, 32]. qPCR on SVZ lysates obtained from adult mice showed no difference between genotypes for any of the genes (Additional file 1: Figure S1A). To investigate the possibility of *Gal-3*^*−/−*^ receiving compensation from other galectins, we carried out qPCR on SVZ samples from *Gal-3*^*+/+*^ and *Gal-3*^*−/−*^ littermates on galectins known to be expressed in the SVZ (Allen Brain Atlas) and [37]. Lgals1, 8, and 9 also showed no difference between genotypes (Additional file 1: Figure S1B). As expected, Lgals3 was absent in *Gal-3*^*−/−*^ mice (*p* < 0.0005).

### Oligodendrocyte-lineage cell density remained stable in the SVZ but decreased in the corpus callosum after cuprizone administration

Microglial cells and inflammation can influence oligodendrogenesis in the SVZ [21]. We therefore investigated the number of Olig2+ oligodendrocyte-lineage cells in the SVZ following cuprizone treatment (Fig. 3f–h) and found that the number of Olig2+ cells remained constant, the SVZ taken as a whole across treatment groups and genotypes (*N* ≥ 6 per group; Fig. 3g; *p* > 0.05 for relevant main effects and interactions in repeated-measures ANOVA). Furthermore, when we separated the SVZ into subregions (dorsolateral, striatal, ventral, septal, and subcallosal), we found there were no differences that occurred within subregions across treatment groups or genotypes (*p* > 0.05 for relevant interactions in repeated-measures ANOVA; data not shown). In contrast to the SVZ, the density of Olig2+ cells in the CC decreased significantly in the 3-week cuprizone treatment groups (Fig. 3h). The density then returned to control levels in the 6-week cuprizone and remyelination treatment groups. This finding is consistent with the spontaneous remyelination that has previously been reported around 5–6 weeks of cuprizone treatment [27].

### Reactive astrocytosis occurred in the SVZ and CC after cuprizone administration

**Fig. 4:**
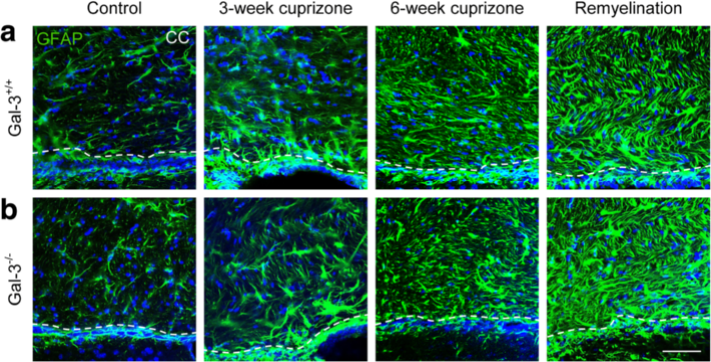
Reactive astrocytosis increases in the SVZ and CC with cuprizone treatment. **a**, **b** Representative images of GFAP in the CC after cuprizone treatment. *Dotted white line* delineates CC from SVZ (note that SVZ was analyzed in separate images of dorsolateral SVZ). *Scale bar* 50 μm

It was important to determine if the reduced number of CD45+ cells in the SVZ neurogenic niche was associated with decreased astrocytosis. GFAP immunoreactivity increased dramatically both in the SVZ and in the CC in the 3-week, 6-week, and remyelination groups but with no obvious qualitative differences between *Gal-3*^*+/+*^ and *Gal-3*^*−/−*^ mice (Fig. 4a, b).

### Proliferation decreased in the SVZ but increased in the CC after cuprizone

**Fig. 5:**
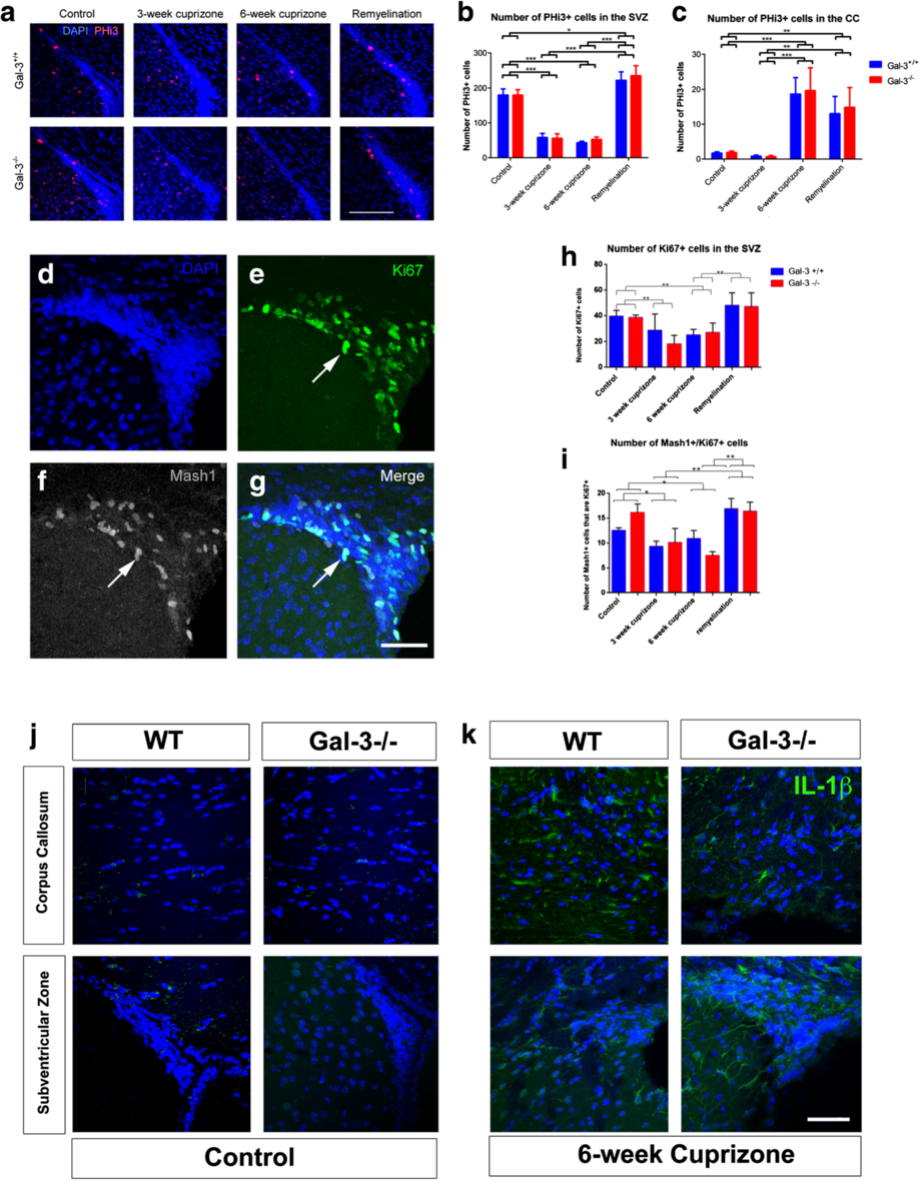
SVZ proliferation decreases after cuprizone treatment. **a** High-magnification images of the dorsolateral SVZ comparing PHi3+ cells in *Gal-3* ^*+/+*^ and *Gal-3* ^*−/−*^ mice across treatment groups. *Scale bar* 100 μm. **b**, **c** Quantification of PHi3+ cells in the SVZ (**b**) and CC (**c**). **d**–**g** Example of Ki67 and Mash1 double immunohistochemistry in the SVZ of a WT mouse in the remyelination group. *Arrow points* to a double-labeled cell. *Scale bar* 50 μm. **h**, **i** Quantification of Ki67+ and Mash1+/Ki67+ cells in the SVZ. Statistics used were one-way ANOVA with Tukey’s post hoc test. Graphs show mean ± SEM. *p < 0.05, **p < 0.01. **j**, **k** Il-1β immunohistochemistry in controls and the 6-week cuprizone group. Note that Il-1β was barely detectable in controls but upregulated in the CC and SVZ in WT and *Gal-3* ^*−/−*^ mice in the 6-week cuprizone group. *Scale bar* 50 μm

Most models of brain disease increase proliferation in the SVZ [38]. Nevertheless, the reduced numbers of Gal-3+ and CD45+ cells in the SVZ could have been associated with reduced proliferation since inflammation can influence SVZ proliferation [20, 21]. We therefore performed immunohistochemistry for PHi3+, which labels proliferating cells in the mitoitc (M) phase of the cell cycle [39]. In the SVZs, we found reduced numbers of PHi3+ cells after 3 and 6 weeks of cuprizone treatment, and they returned to baseline levels in the remyelination group (Fig. 5a, b, Additional file 2: Table S1). In the CC, the number of PH3+ cells increased in the 6-week cuprizone and remyelination groups (*N* ≥ 6 per group; Fig. 5c). Thus, similar to the CD45+ and Gal-3+ cells, the number of Phi3+ cells decreased in the SVZ but increased in the CC.

We previously showed that Gal-3 loss in KO mice did not alter SVZ proliferation [16, 22, 32], and here, the *Gal-3*^*+/+*^ and *Gal-3*^*−/−*^ mice showed virtually the same changes after cuprizone treatment (Fig. 5b, c). We found no significant difference between *Gal-3*^*+/+*^ and *Gal-3*^*−/−*^ mice in the SVZ (*p* = 0.051 for the main effect in ANOVA; *p* = 0.784 for the interaction of genotype with the treatment length in ANOVA) or the CC (*p* = 0.721 for the main effect; *p* = 0.421 for the interaction). To confirm our findings on proliferation and to determine if the decrease was due to loss of transit amplifying progenitor cells, we carried out double immunohistochemistry for the cell cycle marker Ki67 and for the TAP marker Mash1 (Fig. 5d–g, Additional file 3: Figure S2). Compared to controls, the number of Ki67+ cells decreased significantly at the 3- and 6-week time points (Fig. 5h), confirming the PHi3 results. We also found statistically significant decreases in the number of cells that were double positive for Mash1 and Ki67 (Fig. 5i). The number of Ki67+ cells and of Mash1+/Ki67+ cells returned to control levels in the remyelination group. Similar to our data above, we did not observe effects of genotype on KI67+ or Mash1+/Ki67+ cell numbers in the SVZ. These experiments indicate cuprizone treatment significantly reduces SVZ transit amplifying cell proliferation and that they rebound during remyelination.

Cuprizone feeding increases the expression of several genes important for inflammation [40]. Il-1β is upstream of many inflammatory processes, it increases proliferation in multiple cell types, and it drives SVZ neurogenesis [21]. We carried out Il-1β staining and found very little labeling in the CC and SVZ of control mice (Fig. 5j) and in the 3-week group. Il-1β expression in the CC and SVZ increased in the 6-week cuprizone group (Fig. 5k) and in the remyelination group. Whereas there was moderate variablity in immunofluorescence within groups of mice, we did not detect any obvious qualitative differences between Wt and *Gal-3*^*−/−*^ mice at any of the time points (Fig. 5j, k).

**Fig. 6:**
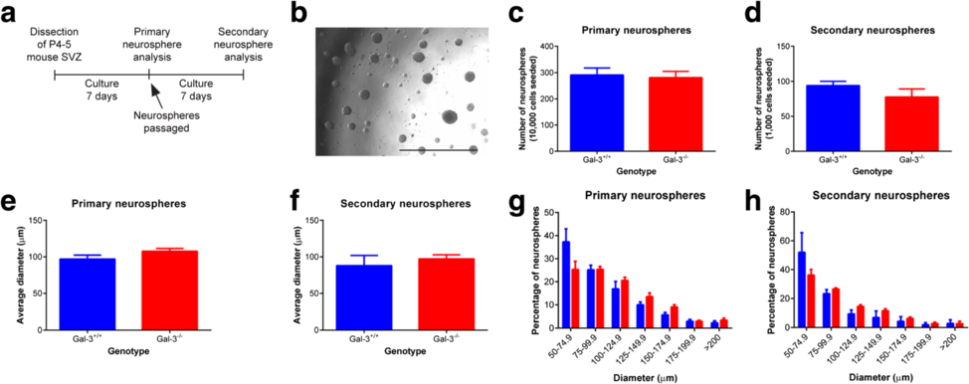
Gal-3 loss does not alter neurosphere proliferation. **a** Diagram showing experimental outline. **b** Sample image of primary neurospheres. *Scale bar* 1 mm. **c**–**h** Graphs comparing *Gal-3* ^*+/+*^ and *Gal-3* ^*−/−*^ mice for number of neurospheres (**c**, **d**), average diameter (**e**, **f**), and distribution of neurosphere sizes (**g**, **h**) for primary (**c**, **e**, and **g**) and secondary (**d, f,** and **h**) neurospheres. Statistics performed using *t* tests with *N* = 5 for all groups except *Gal-3* ^*+/+*^ secondary neurospheres for which *N* = 3. Graphs show mean ± SEM

To verify the lack of difference in proliferation between genotypes, we performed proliferation assays in primary and secondary SVZ neurospheres (Fig. 6). SVZ cells from P4 *Gal-3*^*+/+*^ and *Gal-3*^*−/−*^ mice were cultured for 7 days to produce neurospheres and then passaged and cultured for a further 7 days for analysis (Fig. 6a). Once again, there was no difference between genotypes. The number of neurospheres was consistent for primary neurospheres from *Gal-3*^*+/+*^ compared to *Gal-3*^*−/−*^ mice (*p* = 0.788) and secondary neurospheres (*p* = 0.346) (Fig. 6c, d). The average diameter was consistent for primary and secondary neurospheres (*p* = 0.493; Fig. 6e, f). The distribution of neurosphere sizes was consistent between genotypes for primary and secondary neurospheres (*p* > 0.05 for each comparison; Fig. 6g, h). This in vitro data was consistent with all our in vivo data suggesting SVZ proliferation is not altered in *Gal-3*^*−/−*^ mice.

### Gal-3 absence decreased the number of labeling retaining SVZ cells after cuprizone treatment

**Fig. 7:**
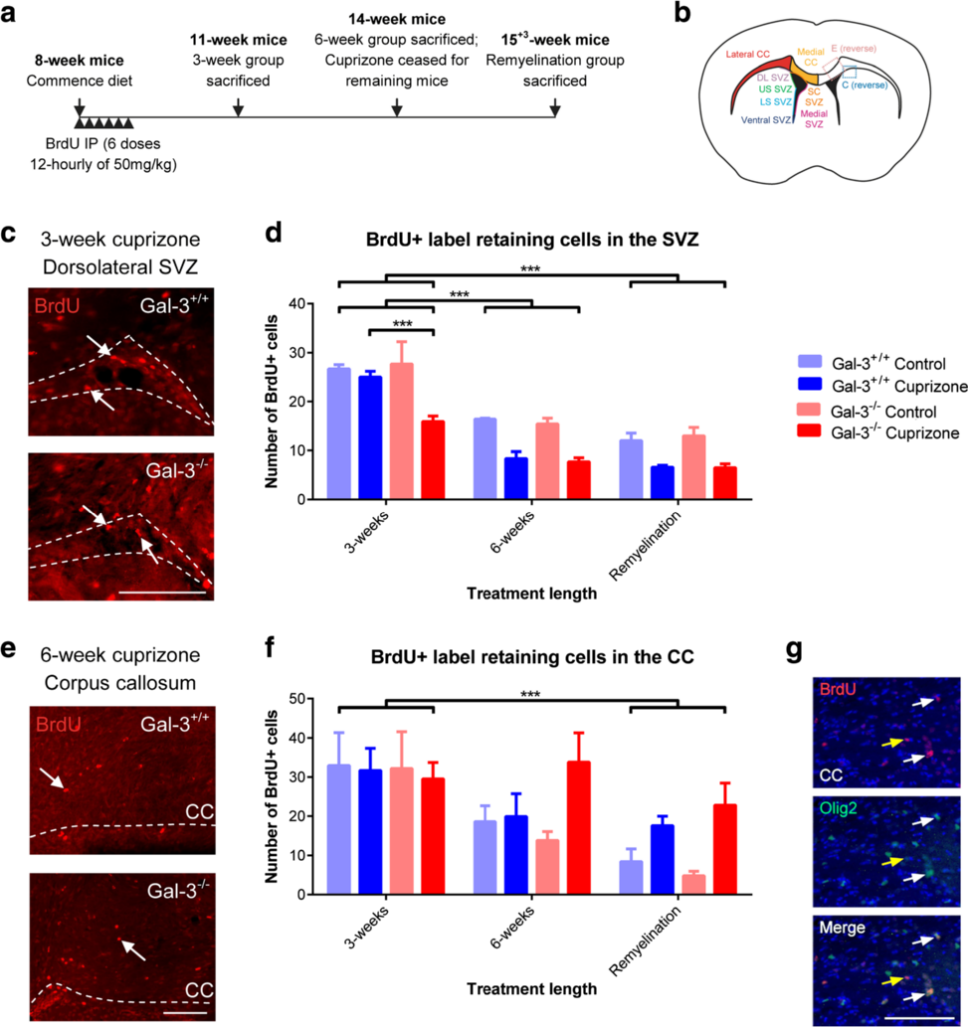
BrdU+ label retaining cells after cuprizone treatment. **a** BrdU regimen administered to mice. **b** Subdivisions of the SVZ and CC that were quantified. **c**, **e** Representative images showing BrdU immunohistochemistry in *Gal-3* ^*+/+*^ and *Gal-3* ^*−/−*^ mice in the dorsolateral SVZ (**c**) and CC (**e**). *White dotted lines* delineate region of interest. *White arrows* show examples of BrdU+ cells. **d**, **f** Graphs showing quantification of BrdU+ cells in the SVZ overall (**d**) and CC overall (**f**). In addition to the statistics shown, cuprizone-treated mice had significantly fewer cells than control mice for all genotype/treatment combinations (*p* ≤ 0.011) except 3-week *Gal-3* ^*+/+*^. **g** Example of BrdU+ cells that were Olig2+ (*white arrows*) and a BrdU+/Olig2- cell (*yellow arrow*). Statistics calculated as repeated-measures ANOVA with *N* ≥ 3. Graphs show mean ± SEM. ***p < 0.005. *Scale bars* 100 μm

We previously reported that loss of Gal-3 promotes SVZ progenitor emigration into the corpus callosum after TMEV injections [16], so we next asked if it also does so after cuprizone. In order to label SVZ cells, we injected BrdU at the beginning of the experiment (Fig. 7a). We first looked for effects with all SVZ and CC subdivisions combined (Fig. 7b). The number of BrdU+ cells left in the SVZ 3 weeks after cuprizone chow was not altered in WT mice (Fig. 7c, d). However, in *Gal-3*^*−/−*^ mice, there was a significant reduction in the number of BrdU+ cells (Fig. 7d). At the 6-week and remyelination time points, both WT and mutant mice had significantly reduced SVZ BrdU+ cell numbers compared to the 3-week group. These data suggest Gal-3 delays the cuprizone-induced emigration of BrdU+ cells. The number of PHi3+ mitotic cells in the CC before cuprizone and at 3 weeks was minimal. However, we found many BrdU+ cells in the CC at 3 weeks which is consistent with them having migrated from the SVZ (Fig. 7e, f). Though more PHi3+ mitotic CC cells were found in the 6-week and remyelination groups, the number of BrdU+ cells decreased at this time point (Fig. 7f). We queried the identity of the emigrated BrdU+ progenitor cells in the CC and found that only a minimal number were positive for the neuroblast marker doublecortin (data not shown). The SVZ generates oligodendrocyte progenitors in demyelinating injuries [13], we therefore stained for Olig2. Across a sample of brains (*N* = 3 per group), we found that 56 % (117 out of 208 cells) of BrdU+ cells were Olig2+ (Fig. 7g).

We next looked for effects in individual SVZ and CC subdivisions (Fig. 7b). With further breakdown using pairwise comparisons, the key difference was fewer BrdU+ cells in the *Gal-3*^*−/−*^ compared to *Gal-3*^*+/+*^ SVZ after 3-week cuprizone specifically in the dorsolateral (*p* = 0.001) and ventral (*p* = 0.006) SVZ (Additional file 4: Table S2). When comparing control and cuprizone mice, there was an increase after cuprizone treatment in the medial CC (*p* = 0.007; Additional file 5: Table S3). The number of cells in the lateral CC did not change significantly from control to cuprizone mice (*p* = 0.852).

### Demyelination occured in the olfactory bulbs after cuprizone treatment

We noticed MBP loss in several brain areas after cuprizone feeding and examined this more carefully in the olfactory bulb (OB), the target structure of SVZ neurogenesis. In the OB, there was extensive loss and then return of MBP expression in the glomerular, external plexiform, mitral cell, and internal plexiform layers (Additional file 6: Figure S3a and b; *N* = 6 per group).

## Discussion

This study showed that the number of Phi3+, Ki67+, CD45+, and Gal-3+ cells decreased in the SVZ after cuprizone treatment which is in contrast to TMEV [16] and most other disease models. The decrease was remarkable since immediately above the SVZ in the CC Phi3+, Ki67+, CD45+, and Gal-3+, cell numbers increased. Because inflammation was decreased in the SVZ, we hypothesized that proliferation would increase in the niche [18]; however, it decreased after cuprizone treatment, suggesting the relationship between inflammation and proliferation in the niche is complex. We also showed that SVZ proliferation was not affected in *Gal-3* knockouts in vivo or in the neurosphere assay. This is in line with our previous work showing that Gal-3 loss does not directly affect SVZ proliferation [16, 22]. However, we labeled SVZ cells with multiple doses of BrdU and found that 3 weeks after cuprizone administration, *Gal-3*^*−/−*^ mice had significantly fewer cells in the SVZ compared to WT controls, suggesting Gal-3 limits emigration of SVZ cells.

The decrease in Gal-3+ SVZ cells was probably not due to the decrease in CD45+ cells since the large majority of SVZ Gal-3 expression is not in CD45+ microglial cells but in SVZ astrocytes and ependymal cells [22]. Interestingly, we showed that cuprizone induced activation of GFAP expression and morphological changes in the SVZ consistent with reactive astrocytosis. Gal-3 expression can increase in striatal astrocytes in response to TMEV [16]. In these experiments, despite the SVZ astrocyte reactivity, Gal-3 expression actually decreased, suggesting Gal-3 is not required for SVZ astrocytic reactivity. Similarly, Gal-3 knockout mice exhibited robust increases in GFAP expression both in the SVZ and the CC. A recent study showed that stab wound injury-induced reactive astrocytosis in the cerebral cortex depends on Gal-3 expression [41]. Thus, it is likely that Gal-3 has different effects on astrocyte reactivity depending on the brain region or type of pathology.

This work adds to the growing list of studies revealing the SVZ as a unique inflammatory niche. We were surprised a number of years ago to find that SVZ microglia are semi-activated in healthy animals [15]. We then made the remarkable observation that traumatic brain injury (TBI) [15] and stroke [32, 42] induce massive inflammation in forebrain parenchyma, but not in the adjacent SVZ. This was in contrast to TMEV infection which was associated with consistent inflammation in the SVZ [17]. In this manuscript, we provide data suggesting that SVZ microglial numbers and inflammation are actually decreased after cuprizone treatment. We have found that Gal-3 is expressed by the same cells in the healthy SVZ as in disease models. It was expressed by astrocytes and ependymal cells homeostatically, after TBI, TMEV, stroke, and cuprizone [16, 22, 32] (and Szele lab unpublished studies). It is particularly surprising that few microglial cells express Gal-3 in the SVZ before or after injury since Gal-3 has been used as a marker of activated macrophages. Taken together, this study and previous work shows that inflammation in the SVZ is tightly regulated and is sensitive to pathological context.

The decrease in markers of inflammation in the SVZ was in sharp contrast with the increased number of Gal-3+ and CD45+ cells in the adjacent corpus callosum. Similar sharp contrasts between indices of inflammation in the SVZ and the adjacent CC, striatum, or cerebral cortex have been noted in TBI and TMEV [15–17]. CD45 labels all cells in the hematopoietic lineage so is insufficient to definitively phenotype them. Here, we found that the number of CD45+ SVZ cells that expressed Iba1 increased slightly after cuprizone. Since Iba1 is associated with microglia, these data suggest that though cuprizone decreased the number of CD45+ SVZ cells, the relative percent of microglia increased. Our previous work found a small number of dendritic cells [17] and T cells [16] in the SVZ; thus, it is possible that some of the CD45+/Iba1-negative cells we found in this study in the SVZ belonged to one of these cell types. Alternatively, a minority SVZ CD45+ microglial cells expressed Iba1 below the level of immunohistochemical detection. It is likely the unique extracellular molecular environment in the SVZ selectively autoregulates inflammation in the niche [16, 43, 44]. We recently demonstrated the SVZ expresses higher levels of chemokine ligands than adjacent regions [16]. A better understanding of how inflammation is regulated in the SVZ may help develop approaches to augment neurogenesis since immune cells cross talk with neural cells and can be either beneficial or detrimental to their homeostatic and reparative functions [45].

Many types of brain injury increase SVZ proliferation [38, 46, 47]; however, activation of neurogenesis depends on the model, species, and level of inflammation. Inflammation was originally shown to dampen adult neurogenesis [18, 19], but recent work has defined multiple modes of microglial activation, some of which increase neurogenesis [20, 21, 45]. Therefore, we were uncertain whether the SVZ phenotype induced by cuprizone described above would be associated with altered proliferation. The demyelination groups had significantly diminished numbers of proliferative SVZ cells and rebounded to control levels in the remyelination group, suggesting a rapid return to homeostasis in the SVZ upon cessation of cuprizone. In contrast to the SVZ, CC proliferation increased at the 6-week and remyelination time points. This paralleled the discrepancy between the SVZ and CC described above and further suggests unique regulation of inflammation in the SVZ.

The proliferative response to cuprizone in the SVZ and the CC was not affected by loss of Gal-3. This was supported by our in vitro data: the number and size of SVZ neurospheres were unaffected by loss of Gal-3. Neurospheres were prepared from P4-5 pups, and it could be that loss of Gal-3 would affect neurosphere growth when prepared from adults. However, loss of Gal-3 did not alter constitutive proliferation in the adult SVZ in three separate murine strains [16, 22]. Similar results were obtained in the MCAO model of stroke—there were no differences between WT and Gal-3 nulls in SVZ or parenchymal proliferation [32]. This is in contrast to a recent study in which loss of Gal-3 inhibited astrocyte proliferation after stab wound injury [41]. Gal-3 binds to a large variety of proteins, has multiple functional effects depending on context, and is frequently expressed de novo after brain injury, together positioning it to have selective effects in different contexts.

One of the key features of SVZ progenitors is their capacity for long-distance migration to the OB and emigration towards injured regions of the brain. There is good evidence that SVZ progenitors are beneficial by a combination of neural replacement and neuroprotection [48, 49]. To track potential SVZ progenitor emigration, we labeled cells with BrdU at the beginning of cuprizone administration. The large majority of cells that are proliferative and get labeled by BrdU in the forebrain before damage are SVZ cells. Therefore, BrdU+ cells outside of the SVZ at later time points could be inferred to have migrated from the SVZ. Interestingly, we showed decreased BrdU+ cell numbers in the SVZ but increased numbers in the CC after cuprizone. At 3 weeks, the number of BrdU+ cells that were gone from the SVZ was significantly greater in *Gal-3*^*−/−*^ than in WT mice, suggesting that Gal-3 normally inhibits emigration. This is supported by our previous finding that lack of Gal-3 causes SVZ progenitors to switch migration from their rostral direction to a local exploratory motility as a prelude to emigration [22]. It is also consistent with our TMEV demyelination model wherein loss of Gal-3 increased SVZ progenitor emigration [16]. These data are consistent with cuprizone inducing BrdU+ cells to migrate from the SVZ to the CC.

Because of the decreased rostral migration previously observed in *Gal-3*^*−/−*^ mice [22], the decreased number of BrdU+ cells in the SVZ of *Gal-3*^*−/−*^ mice in this study was probably not caused by greater rostral migration towards the OB. We demonstrated decreased MBP immunofluorescence in the OB of WT mice after cuprizone, suggesting demyelination. However, we do not know if OB demyelination was increased in *Gal-3*^*−/−*^ mice relative to WTs, which could have increased rostral migration and explained the decreased numbers of BrdU+ cells in the SVZ at 3 weeks post-cuprizone. Using immunohistochemistry for Gal-3 and CD45, we previously noticed that cuprizone and TMEV caused inflammation in multiple forebrain regions [17], such as the OB. We have other data suggesting OB demyelination after TMEV based on reduced MBP immunofluorescence (Szele lab, unpublished observations). The extent of OB and olfactory tract demyelination in MS and neuromyelitis optica patients is correlated with disease severity [50]. We propose the cuprizone and TMEV-IDD animal models will help elucidate the pathophysiological mechanisms and functional consequences of OB inflammation and demyelination in MS.

The majority of cells that emigrated from the SVZ were likely in the oligodendrocyte lineage as many BrdU+ labeled cells in the CC were Olig2+. There is ample evidence that human MS and models of demyelination induce SVZ-derived oligodendrocytes to emigrate [10–13, 51]. In this study, the number of Olig2+ cells in the CC decreased at 3 weeks post-cuprizone and then increased suggesting cuprizone initially killed oligodendrocytes which were then replaced by SVZ-derived oligodendrocyte precursors. Recent work has suggested Gal-3 alters the rate of oligodendrocyte maturation and extent of demyelination [26]. We did not find any evidence for differences in Olig2+ cells in the SVZ or CC between WT and *Gal-3*^*−/−*^ mice. We also examined the expression of the mature oligodendrocyte marker CC1 [35]. We did not find that the percent of Olig2+ cells that expressed CC1 differed between Wt and Gal-3^*−/−*^ mice. Both genotypes also exhibited an increased percent of Olig2+ that were CC1+ in the remyelination group. These results suggest Gal-3 loss does not affect oligodendrocyte differentiation. Finally, although SVZ neuroblast migration towards demyelination has been documented [52], we found very few BrdU+ cells in the CC that expressed the immature neuronal marker Dcx.

A study by Hoyos and colleagues showed that loss of Gal-3 inhibited remyelination in the CC after cuprizone as measured by MBP, suggesting that Gal-3 normally has a positive effect on remyelination [27]. Similar to that study, we showed loss of MBP immunofluorescence in the CC in the 6-week post-cuprizone group suggesting demyelination. However, we did not find that loss of Gal-3 affected MBP loss or return to normal values in the CC. We are not certain what accounts for this discrepancy; however, it is important to note that Hoyos et al. performed experiments in a different Gal-3 knockout mouse, which lacks exon V [27], whereas the mouse used here lacks exons II, III, and IV [53].

## Conclusions

We provide further evidence in this study that inflammation in the SVZ is uniquely regulated. Compared to the recently published TMEV model of severe inflammation and demyelination [16], the effects of Gal-3 are more subtle in the milder cuprizone demyelination. Nevertheless, we did show here that loss of Gal-3 is associated with increased SVZ progenitor emigration to demyelinated regions. Since Gal-3 is druggable, our combined studies suggest clinical trials of molecules that modulate Gal-3 function may be warranted.

## Abbreviations

ANOVA, analysis of variance; BrdU, 5-bromo-2′-deoxyuridine; CC, corpus callosum; CD45, complement of differentiation 45; Dcx, doublecortin; Gal-3, galectin-3; GFAP, glial fibrillary acidic protein; Iba1, ionized calcium-binding adapter molecule 1; LV, lateral ventricle; MBP, myelin basic protein; MS, multiple sclerosis; OB, olfactory bulb; Olig2, oligodendrocyte lineage transcription factor 2; PHi3, phosphohistone 3; qPCR, quantitative polymerase chain reaction; RMS, rostral migratory stream; SEM, standard error of the mean; SVZ, subventricular zone; TAP, transit amplifying progenitor

## Additional files

Additional file 1: Figure S1. Gal-3 absence does not alter candidate gene expression. A: Graph shows the relative expression of the candidate genes Ccl2, Ccr2, Egfr, and Mmp9 in the SVZ of *Gal-3* ^*+/+*^ and *Gal-3* ^*−/−*^ mice. It demonstrates fold change compared to *Gal-3* ^*+/+*^ mice for quantities normalized to the housekeeping gene B2m. Graph shows mean ± SEM. Graph demonstrates fold change compared to *Gal-3* ^*+/+*^ mice. Statistics calculated as *t* test using SPSS. No significant differences found. B: Gal-3 absence is not compensated by other galectins. Graph shows qPCR comparison of SVZ galectin expression in *Gal-3* ^*+/+*^ and *Gal-3* ^*−/−*^ mice. It demonstrates fold change compared to *Gal-3* ^*+/+*^ mice for quantities normalized to the housekeeping gene B2m. Genes include Lgals1, 3, 8, and 9, which correspond to Gal-1, 3, 8, and 9, respectively. Statistics calculated as *t* test with *N* = 3 samples per genotype, each sample containing pooled SVZs from four mice. Graph shows mean ± SEM. ****p* < 0.001. (TIF 9099 kb) Additional file 2: Table S1. PHi3+ cells int he SVZ and CC after cuprizone treatment. (JPG 380 kb) Additional file 3: Figure S2. Ki67 and Mash1 immunohistochemistry in the SVZ. Representative examples of Ki67 and Mash1 immunohistochemistry in the SVZ. Scale bar 50 μm. (TIF 5734 kb) Additional file 4: Table S2. Pairwise comparisons for genotype within region*length*genotype interaction in the SVZ. (JPG 288 kb) Additional file 5: Table S3. Pairwise comparison for region*treatment in the CC. (JPG 127 kb) Additional file 6: Figure S3. Cuprizone causes loss of MBP expression in the olfactory bulb. A and B: OB sections with MBP immunohistochemistry from WT mice. Dotted box shows region of images in B. GL: glomerular layer, EPL: external plexiform layer, MCL/IPL: mitral cell layer/internal plexiform layer, GrL: granular layer. Scale bars 500 mm (A) and 100 mm (B). C: Graphs showing percentage of pixels with fluorescence intensity above threshold in the glomerular, external plexiform, and granular layers. Statistics calculated as repeated-measures ANOVA with *N* = 6. Graphs show mean ± SEM. **p* ≤ 0.05, ***p* ≤ 0.01, ****p* ≤ 0.001. (TIF 18577 kb)
