# Supplementary figures and images for: Cuprizone demyelination induces a unique inflammatory response in the subventricular zone
Source: J Neuroinflammation. 2016 Aug 22;13(1):190. doi: 10.1186/s12974-016-0651-2 (PMC4994223; doi:10.1186/s12974-016-0651-2)

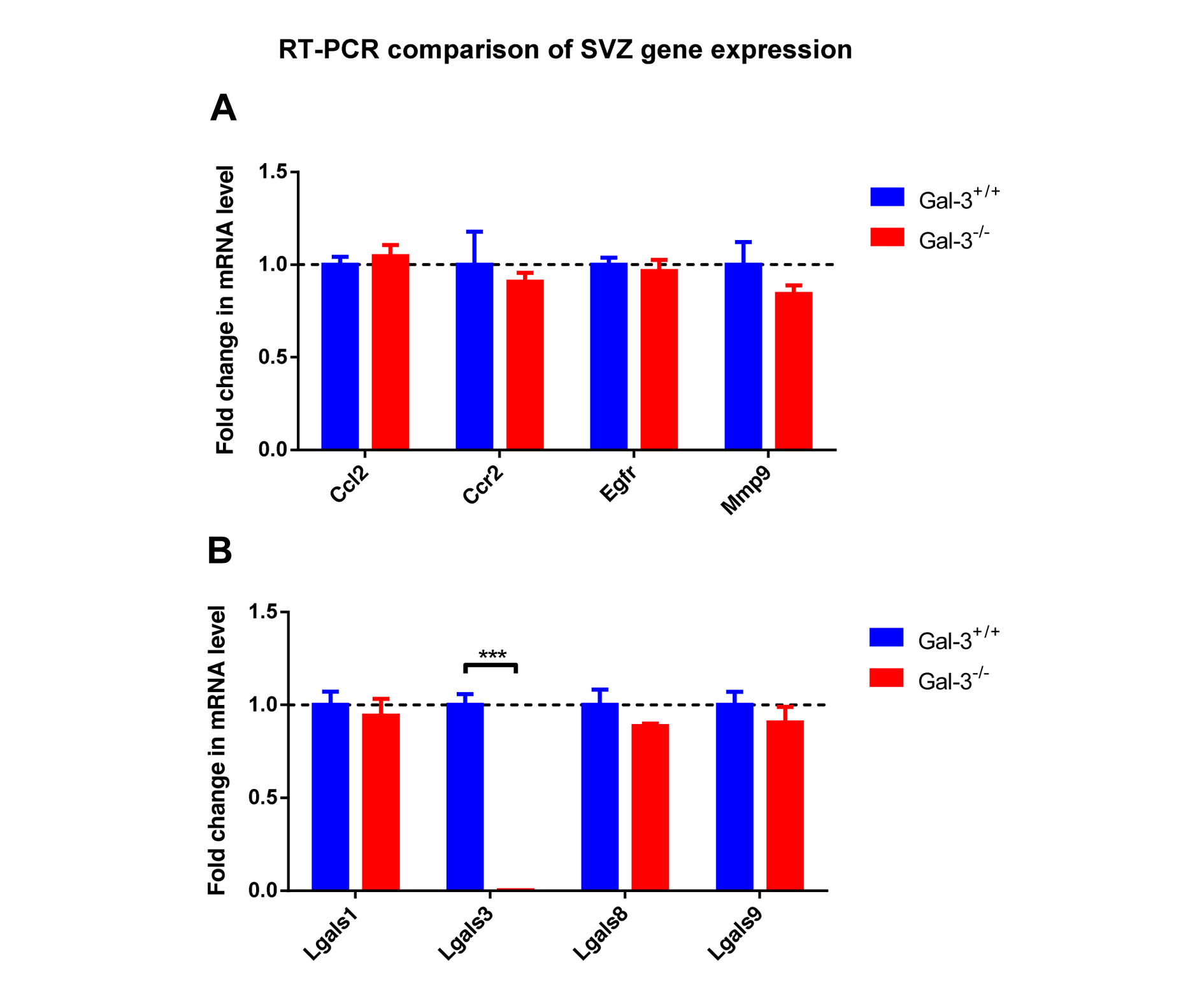

Supplement: Additional file 1: Figure S1. — Gal-3 absence does not alter candidate gene expression. A: Graph shows the relative expression of the candidate genes Ccl2, Ccr2, Egfr, and Mmp9 in the SVZ of Gal-3 +/+ and Gal-3 −/− mice. It demonstrates fold change compared to Gal-3 +/+ mice for quantities normalized to the housekeeping gene B2m. Graph shows mean ± SEM. Graph demonstrates fold change compared to Gal-3 +/+ mice. Statistics calculated as t test using SPSS. No significant differences found. B: Gal-3 absence is not compensated by other galectins. Graph shows qPCR comparison of SVZ galectin expression in Gal-3 +/+ and Gal-3 −/− mice. It demonstrates fold change compared to Gal-3 +/+ mice for quantities normalized to the housekeeping gene B2m. Genes include Lgals1, 3, 8, and 9, which correspond to Gal-1, 3, 8, and 9, respectively. Statistics calculated as t test with N = 3 samples per genotype, each sample containing pooled SVZs from four mice. Graph shows mean ± SEM. ***p < 0.001. (TIF 9099 kb) [file 12974_2016_651_MOESM1_ESM.tif]

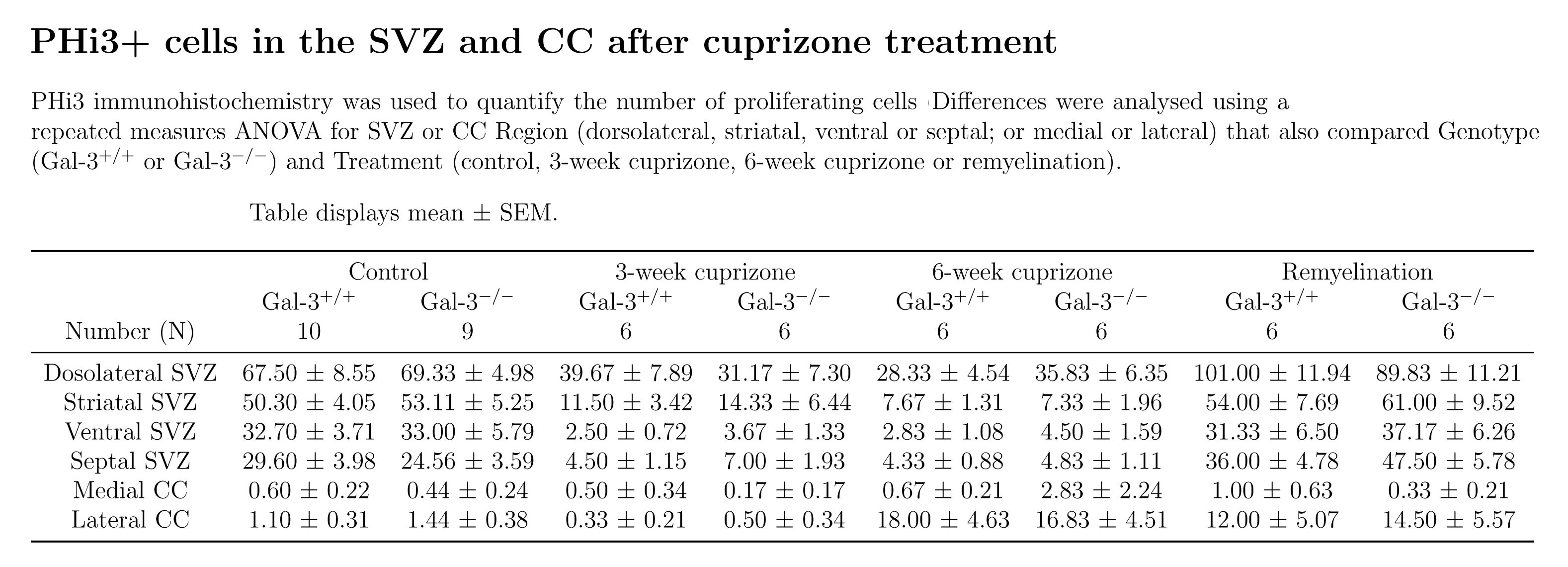

Supplement: Additional file 2: Table S1. — PHi3+ cells int he SVZ and CC after cuprizone treatment. (JPG 380 kb) [file 12974_2016_651_MOESM2_ESM.jpg]

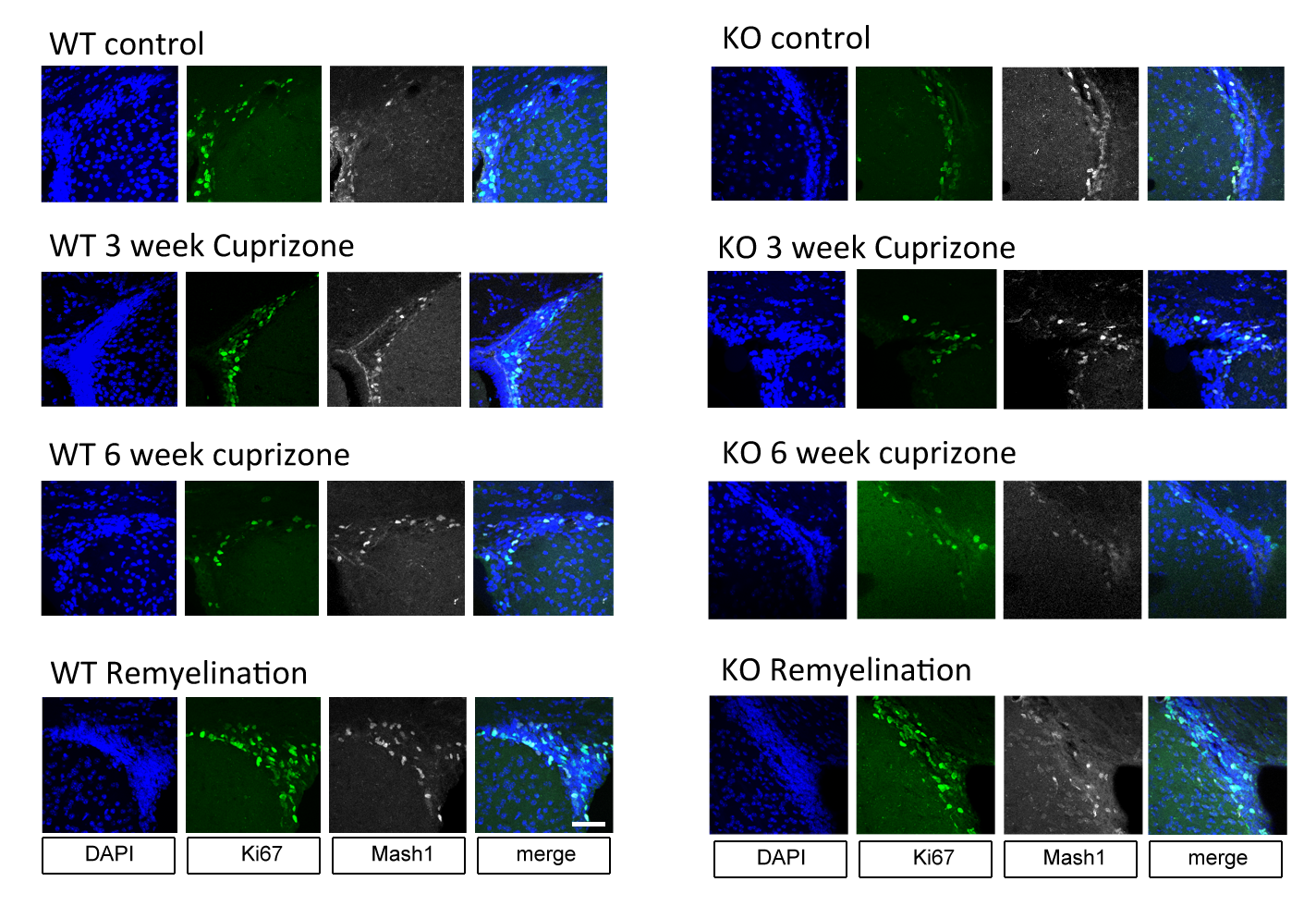

Supplement: Additional file 3: Figure S2. — Ki67 and Mash1 immunohistochemistry in the SVZ. Representative examples of Ki67 and Mash1 immunohistochemistry in the SVZ. Scale bar 50 μm. (TIF 5734 kb) [file 12974_2016_651_MOESM3_ESM.tif]

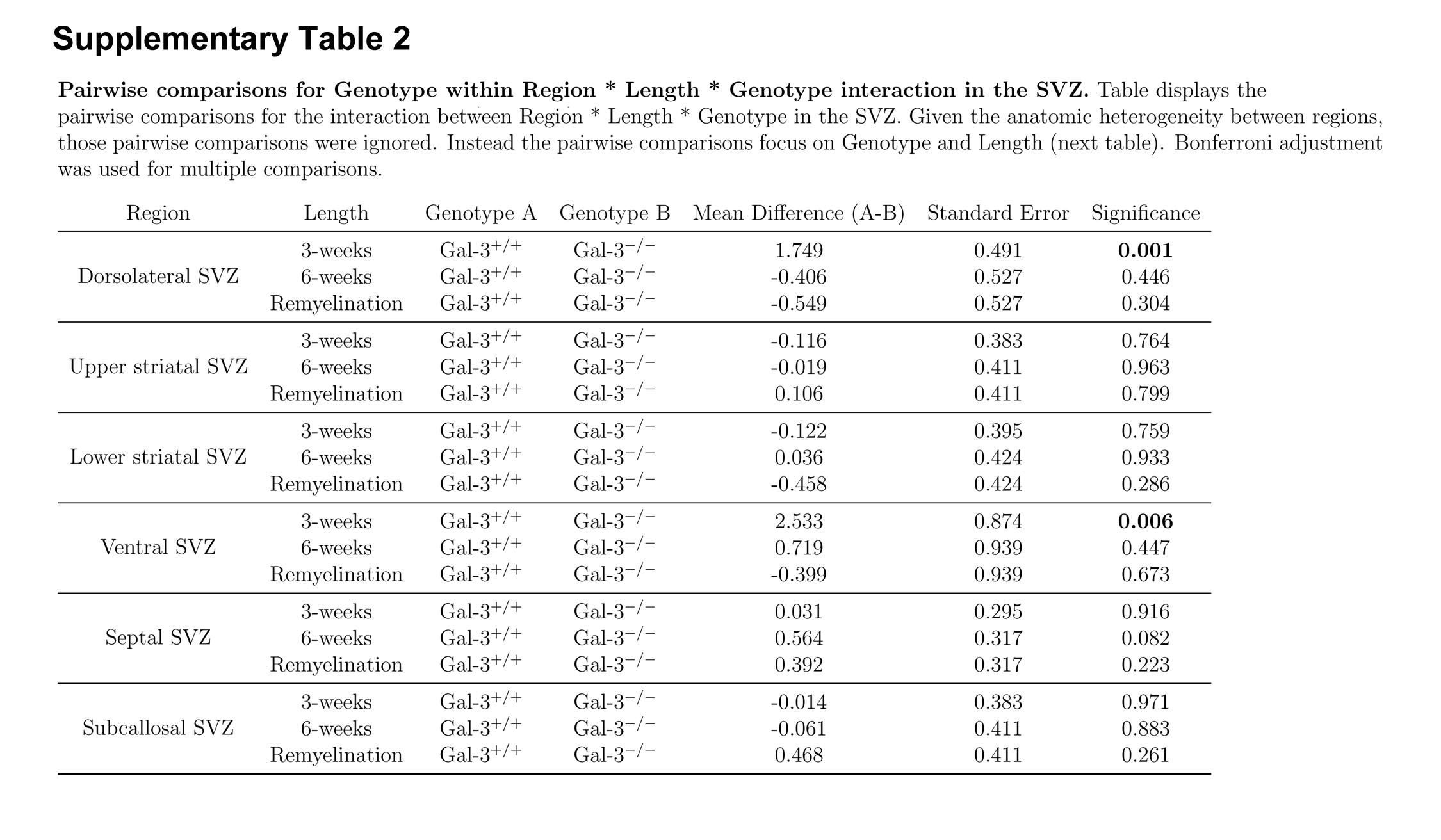

Supplement: Additional file 4: Table S2. — Pairwise comparisons for genotype within region*length*genotype interaction in the SVZ. (JPG 288 kb) [file 12974_2016_651_MOESM4_ESM.jpg]

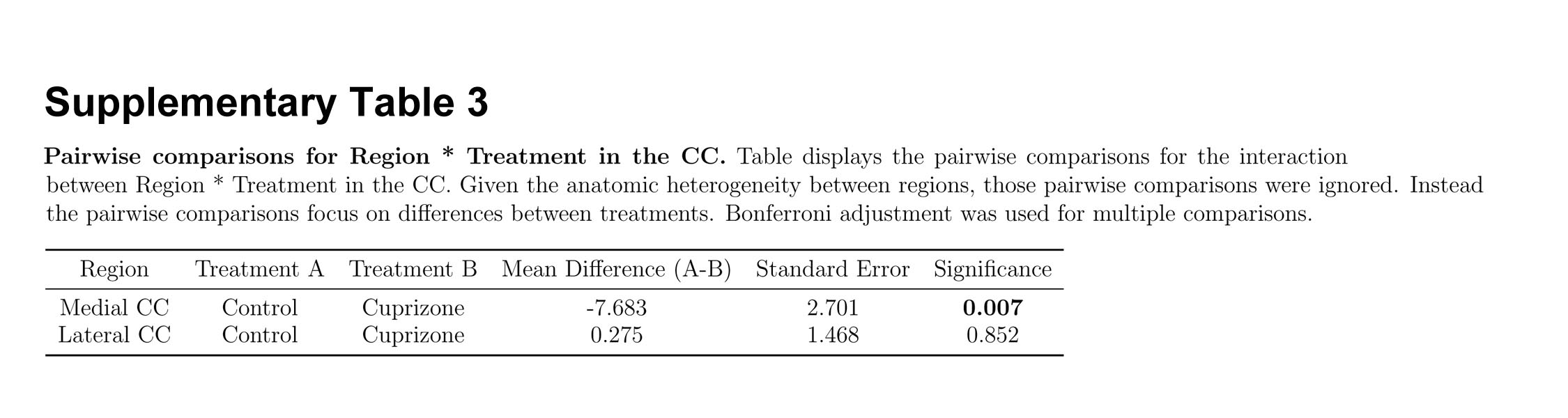

Supplement: Additional file 5: Table S3. — Pairwise comparison for region*treatment in the CC. (JPG 127 kb) [file 12974_2016_651_MOESM5_ESM.jpg]

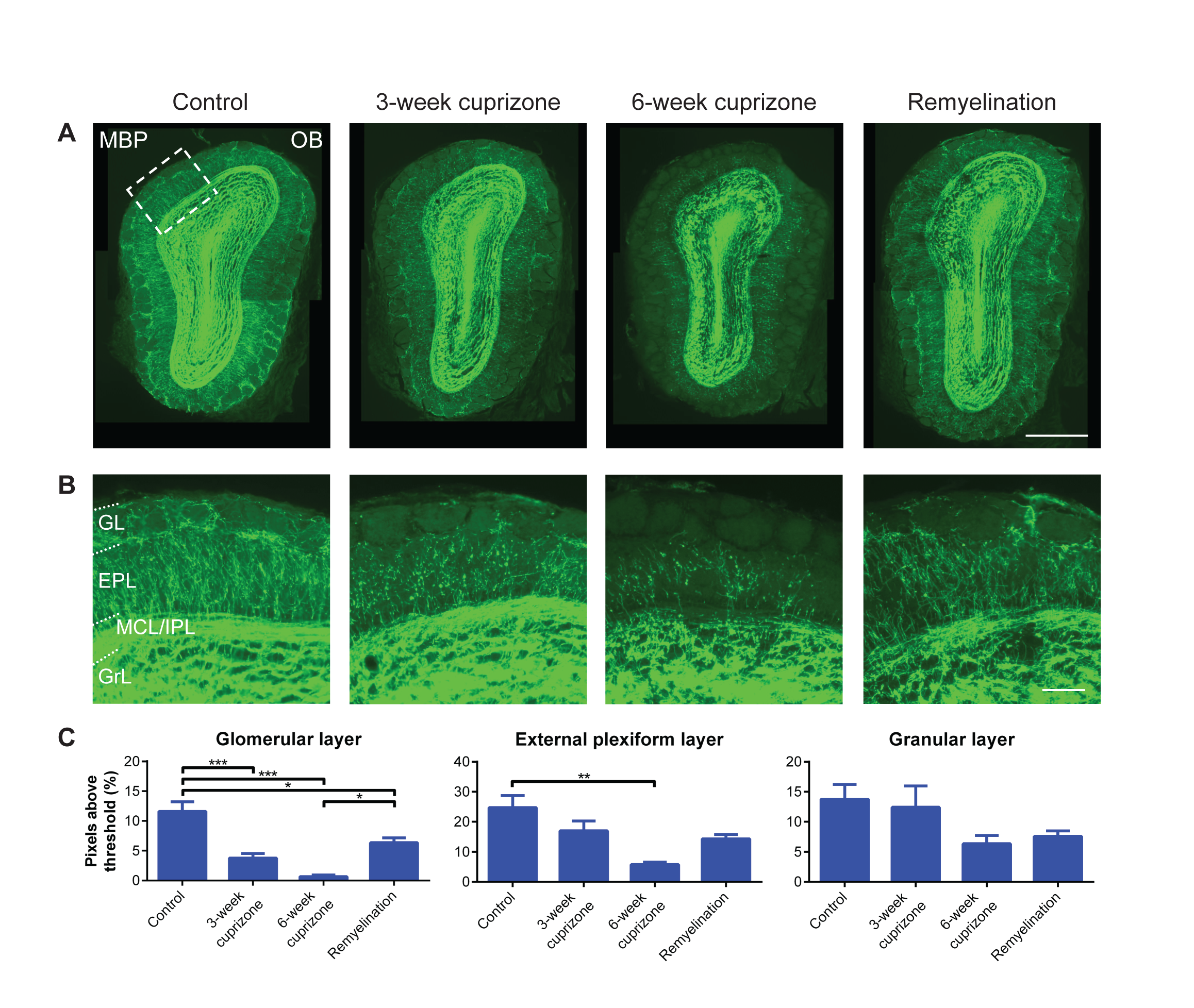

Supplement: Additional file 6: Figure S3. — Cuprizone causes loss of MBP expression in the olfactory bulb. A and B: OB sections with MBP immunohistochemistry from WT mice. Dotted box shows region of images in B. GL: glomerular layer, EPL: external plexiform layer, MCL/IPL: mitral cell layer/internal plexiform layer, GrL: granular layer. Scale bars 500 mm (A) and 100 mm (B). C: Graphs showing percentage of pixels with fluorescence intensity above threshold in the glomerular, external plexiform, and granular layers. Statistics calculated as repeated-measures ANOVA with N = 6. Graphs show mean ± SEM. *p ≤ 0.05, **p ≤ 0.01, ***p ≤ 0.001. (TIF 18577 kb) [file 12974_2016_651_MOESM6_ESM.tif]
